# Expert Consensus on Optimizing the Strategy for the Prevention of Vitamin D Deficiency in Central Asia: From Scientific Evidence to Real-World Practice

**DOI:** 10.3390/nu18132122

**Published:** 2026-06-30

**Authors:** Pawel Pludowski, Larisa Makalkina, Rimma Bazarbekova, Ainur Dossanova, Galymzhan Togizbayev, Gulzhan Gabdulina, Galina Grebennikova, Assel Jaimbetova, Dilrabo Kayumova, Sevara Irgasheva, Gulnaz Bobushova

**Affiliations:** 1 Department of Clinical Biochemistry, The Children’s Memorial Health Institute, 04-730 Warsaw, Poland; 2 Department of Clinical Pharmacology, NJSC “Astana Medical University”, Astana 010000, Kazakhstan; 3 Department of Endocrinology, Non-Profit Educational Organization “Kazakh-Russian Medical University”, Almaty 050004, Kazakhstan; 4 Research Institute of Cardiology and Internal Diseases, Almaty 050000, Kazakhstan; 5 Department of General Practitioner’s Diseases, NJSC “S.D. Asfendiyarov Kazakh National Medical University”, Almaty 050012, Kazakhstan; 6 IRM Clinic, Almaty 050012, Kazakhstan; 7 Department of Obstetrics and Gynecology, Tashkent State Medical University, Tashkent 100109, Uzbekistan; kdilrabo@mail.ru; 8 Republican Specialized Scientific and Practical Medical Center for Maternal and Child Health, Ministry of Health of the Republic of Uzbekistan, Tashkent 100140, Uzbekistan; 9 Department of Family Medicine, Kyrgyz Russian-Slavic University Named After B.N. Yeltsin, Bishkek 720000, Kyrgyzstan

**Keywords:** vitamin D, 25-hydroxyvitamin D, vitamin D deficiency, vitamin D insufficiency, hypovitaminosis D, avitaminosis D, prevention, cholecalciferol, Central Asia

## Abstract

Background/Objectives: Vitamin D deficiency and insufficiency represent a widespread problem in the majority of Central Asian countries, attributable to the geographical location of the region, urbanization, and dietary patterns of the population. Given that vitamin D not only participates in the regulation of calcium and phosphate metabolism but also exerts pleiotropic effects on various organs and systems, its insufficiency and deficiency are associated with a broad spectrum of pathological conditions, ranging from asymptomatic manifestations to severe clinical symptoms, including the development of autoimmune diseases, metabolic disorders, cardiovascular, gynecological and reproductive, and rheumatological conditions. The development of national and interdisciplinary guidelines addressing the diagnosis, prevention of insufficiency, and correction of vitamin D deficiency in the countries of Central Asia represents an important step toward the establishment of effective preventive programs and treatment strategies, which may contribute to a reduction in the prevalence of diseases associated with vitamin D deficiency. The aim of the present work is to formulate a resolution capturing the conclusions and recommendations derived from an interdisciplinary expert discussion. Methods: An Expert Council meeting with the participation of specialists in endocrinology, obstetrics and gynecology, rheumatology, clinical pharmacology, and other medical disciplines from Central Asian and European countries was conducted in Almaty (Kazakhstan) on 18 June 2025. During this meeting, the pleiotropic action of vitamin D was extensively discussed basing on RCTs and observational studies. Results: Following the Expert Council meeting, current international clinical guidelines, scientific research data, and relevant epidemiological evidence were reviewed, leading to the formulation of a resolution that reflects the agreed-upon recommendations for the prevention of vitamin D insufficiency and the correction of vitamin D deficiency across different age groups according to baseline vitamin D metabolite levels. Conclusions: The expert discussion emphasized the need for unified interdisciplinary approaches to the diagnosis, correction, and prevention of vitamin D deficiency in the countries of Central Asia. The proposed recommendations may serve as a foundation for the development of national clinical protocols and the implementation of effective preventive and therapeutic strategies in the countries of Central Asia.

## 1. Introduction

In Kazakhstan and the countries of Central Asia, there are currently no national or interdisciplinary guidelines regulating the diagnosis, correction, and prevention of vitamin D deficiency and insufficiency, which necessitates the creation of a working group comprising experts in endocrinology, rheumatology, obstetrics and gynecology, clinical pharmacology, and family medicine to conduct expert discussions. The outcome of the Expert Council meeting was the identification of priorities for developing unified and harmonized approaches to managing patients with vitamin D deficiency and for formulating prevention strategies for different population categories.

Over the past decades, vitamin D has become the subject of numerous scientific studies in the field of medicine and public health, largely due to the discovery of vitamin D receptors in a wide range of human organs and tissues [1,2]. In addition to its well-known key role in the regulation of calcium and phosphate metabolism, vitamin D, acting as a prohormone, exhibits pleiotropic effects and has a significant impact on various metabolic processes. It also demonstrates anti-inflammatory and antifibrotic properties, underscoring its importance in the prevention and treatment of a wide spectrum of diseases [1,2,3,4,5,6].

Vitamin D insufficiency is a condition in which the concentration of 25(OH)D in the blood is below the optimal level (30–50 ng/mL), at which subclinical disturbances in bone tissue mineralization and an increased risk of developing various chronic diseases may occur. Vitamin D deficiency is characterized by a more pronounced decrease in 25(OH)D concentrations (below 20 ng/mL) and is accompanied by clinically significant disorders. Both insufficiency and deficiency of vitamin D are associated with a wide range of pathological conditions, varying from asymptomatic manifestations to severe clinical symptoms, including the development of autoimmune diseases, metabolic syndrome, obesity, depression, reduced fertility, miscarriage, menstrual irregularities, aggravation of menopausal symptoms, and progression of osteoporosis in postmenopausal women. A number of studies have demonstrated an association between low vitamin D levels and an increased risk of socially significant diseases such as cancer, cardiovascular diseases, osteoporosis and hyperparathyroidism, diabetes mellitus, infectious and autoimmune diseases, depression, infertility, endometriosis, and polycystic ovary syndrome (PCOS) [7,8,9,10,11,12,13,14].

In Kazakhstan, as well as in other countries of Central Asia, a high prevalence of vitamin D deficiency has been observed [15]. Firstly, this is associated with the geographical location of these countries. According to the literature, populations living north of 35–40° north latitude (N lat.) are at high risk of vitamin D deficiency [16,17]. Geographically, Kazakhstan extends from the 40th parallel north at its southernmost point to the 56th parallel north at its northernmost point, while the southern regions of Central Asia (Turkmenistan, the southern part of the Republic of Uzbekistan, and Tajikistan) are located between the 35th and 40th parallels north, which is considerably farther from the equator. Secondly, the traditional diet in the Central Asian region is characterized by limited consumption of key sources of vitamin D, such as fish [18,19,20,21,22]. In addition, the absence of large-scale food fortification further aggravates the problem of insufficient dietary intake of vitamin D. Thirdly, urbanization and modern lifestyle factors lead to reduced synthesis of vitamin D in the skin due to limited sun exposure and decreased penetration of UVB rays through polluted air [23,24,25].

Thus, given the geographical, climatic, and social characteristics of the Central Asian region, the problem of vitamin D deficiency requires special attention. The development of unified approaches to identifying risk groups, as well as to establishing diagnostic and correction algorithms and prevention strategies for vitamin D insufficiency, will form the foundation for creating national clinical guidelines that ensure interdisciplinary coordination among physicians of various specialties.

## 2. Methods

The expert panel included 11 participants with expertise in vitamin D from tertiary centers across Central Asia and from Central Europe. All panelists participated in each “e-mail” round and the final face-to-face meeting, and all are authors of this manuscript. An executive writing group (L.M.; R.B.; A.D.; G.T.; G.G. (Gulzhan Gabdulina); G.G. (Galina Grebennikova); A.J.; D.K.; S.I; G.B. and P.P.) was appointed to prepare the paragraphs of draft of the manuscript, according to their medical specialties (endocrinology, obstetrics and gynecology, rheumatology, clinical pharmacology, and other medical disciplines). This draft manuscript was then sent to an expert group for critical revision. This document provides the consensus agreement of a joint expert panel and a working group with contributors, expert clinicians and researchers focused on vitamin D and its associations with major health problems, global health, epidemiology of the deficiency, and relations to human morbidity. Agreement on established recommendations was achieved after extensive and comprehensive discussions and revisions of the document, leading to a consensus on all items.

More precisely, in preparation for the Expert Council meeting and the development of the consensus document, the expert group conducted a structured literature review. The search was performed in Scopus, Google Scholar, PubMed/MEDLINE, and Web of Science databases. The search covered publications from 2010 to 2025. The following keywords and their combinations were used: “vitamin D,” “vitamin D deficiency,” “vitamin D insufficiency,” “prevention of vitamin D insufficiency,” “prevention of vitamin D deficiency,” “treatment of vitamin D deficiency,” “cholecalciferol,” “25-hydroxyvitamin D,” “25(OH)D,” “vitamin D supplementation,” and “Central Asia.” Additional searches were performed using regional terms, including “Kazakhstan,” “Uzbekistan,” “Kyrgyzstan,” “Tajikistan,” “Turkmenistan,” and “Central Asia,” in combination with “vitamin D deficiency,” “vitamin D insufficiency,” and “25(OH)D.” The reviewed sources included international clinical guidelines, expert consensus statements, systematic reviews, randomized controlled trials, observational studies, and available regional epidemiological publications. The review of available evidence was used to the development of initial consensus statements and to support expert discussion during the preparation of the resolution. This work was designed as an expert consensus document rather than a systematic review or meta-analysis; therefore, the literature review was intended to provide an evidence-informed basis for practical regional recommendations. Of note, the process, similar to the Delphi methodology, comprised three phases: preparation, two rounds of online exchange of e-mails (as a part of discussion on our votes), and a final face-to-face consensus meeting. The panel members decided to refrain from grading the strength of the recommendations; thus, no quantitative rating of the evidence was used. Statements reaching the overall consensus were accepted; remaining items were further discussed at an in-person meeting in Almaty on 18 June 2025 (Kazakhstan), attended by all panelists. Revised phrases were again discussed and finally accepted by all participants, achieving consensus for all final statements for clinical practice of Central Asia on prevention and treatment of vitamin D deficiency.

## 3. Epidemiology of Vitamin D Deficiency

The results of numerous international studies indicate a high prevalence of vitamin D deficiency worldwide [16,26,27]. At the same time, epidemiological data for the countries of Central Asia remain limited; however, the available studies demonstrate a significant prevalence of vitamin D deficiency even under the relatively warm climatic conditions of the region. In 2024, the results of a systematic review and meta-analysis on the prevalence of vitamin D deficiency in Kazakhstan were published [28]. The analysis included seven studies conducted in various regions of the country, including the cities of Almaty, Astana, and Karaganda, as well as the Almaty region [15,29,30,31,32,33,34]. All studies were published between 2015 and 2024 and collectively included 3616 participants, among whom 2239 were found to have vitamin D deficiency [28]. The prevalence of vitamin D deficiency was as follows: among healthy adults—55% (95% CI: 38–70%), among individuals with various chronic diseases—60% (95% CI: 38–79%), and in the general population—57.2% (95% CI: 49.2–64.8) [28]. In four out of five studies, the mean serum 25(OH)D concentration was below 20 ng/mL, which correlates with data from the global literature and confirms the high prevalence of vitamin D deficiency in countries of the Northern Hemisphere, particularly in populations with low fish consumption [29,30,31,32]. In another cross-sectional study conducted in Kazakhstan, vitamin D deficiency was identified in more than 65% of the population [15]. Women of reproductive age (70%) and pregnant women (75%) were found to be particularly vulnerable groups [15]. Similar trends have been identified in neighboring countries of Central Asia. According to Buyuklyanov A.A. et al., in the Kyrgyz Republic, vitamin D deficiency and insufficiency were recorded in 53% of the examined individuals [35]. In Uzbekistan, according to Akhmedova D. et al., vitamin D deficiency and insufficiency were diagnosed in 82% of young children [36]. Among adolescents and adults (aged 11–84 years) residing in Tashkent, only 17.3% of participants had normal 25(OH)D concentrations; 51.7% showed insufficiency, and 29.1% had deficiency [37]. Moreover, vitamin D deficiency was found in 100% of women with symptomatic uterine fibroids and in 94% of women with asymptomatic fibroids [38]. According to Kayumova D.T. et al., vitamin D deficiency and marked insufficiency are observed in 100% of women with symptomatic uterine fibroids and in 94% of those with asymptomatic fibroids, highlighting the potential role of vitamin D hypovitaminosis in the pathogenesis of uterine fibroids and in the severity of their clinical manifestations [38]. Thus, the research data indicate that vitamin D deficiency in the countries of Central Asia is widespread, affecting various age and social groups. This underscores the need for further epidemiological studies, as well as the development of national prevention strategies and interdisciplinary clinical approaches to the correction of vitamin D deficiency.

## 4. Metabolism of Vitamin D in the Human Body

Vitamin D is a fat-soluble secosteroid that enters the body either as cholecalciferol (vitamin D_3_) synthesized in the skin under exposure to UVB radiation, or as ergocalciferol (vitamin D_2_) obtained from plant-based foods or from fortified food products and dietary supplements (DS). Both forms of vitamin D are biologically inactive and require sequential biotransformation within the body.

### 4.1. Hepatic Stage

In the liver, cholecalciferol and ergocalciferol are hydroxylated to form 25-hydroxyvitamin D (calcidiol/calcifediol), which is the main circulating metabolite that can be assayed in laboratory. The concentration of calcidiol/calcifediol in the blood reflects vitamin D status and is used in clinical practice to diagnose its deficiency and insufficiency.

### 4.2. Renal Stage

In the kidneys, calcidiol/calcifediol undergoes a second hydroxylation to form 1,25-dihydroxyvitamin D_3_ (calcitriol), which represents the active form of vitamin D_3_. Calcitriol influences not only calcium and phosphate metabolism but also exerts immunomodulatory, anti-inflammatory, and antiproliferative effects. The metabolic activation pathway for vitamins D_2_ and D_3_ is identical; however, vitamin D_3_ has greater stability, bioavailability, and is more effective in increasing 25(OH)D concentration values. Therefore, it is the form most commonly used in medicinal products and dietary supplements.

## 5. Skeletal Effects of Vitamin D

The role of vitamin D as a regulator of calcium, phosphate, and mineral metabolism is well established [8]. The active form of vitamin D calcitriol (1,25(OH)_2_D_3_) enhances intestinal absorption of calcium and phosphorus through specific transport proteins, promotes their reabsorption in the kidneys, and ensures mineralization of the bone matrix. Calcitriol participates in bone matrix mineralization by regulating the activity of osteoblasts and osteoclasts, thereby maintaining the balance between bone formation and resorption processes. In addition, vitamin D regulates and suppresses the secretion of parathyroid hormone, preventing the development of secondary hyperparathyroidism and loss of bone mass.

In vitamin D deficiency, bone mineralization is impaired, leading to the development of rickets in children and osteomalacia in adults, as well as an increased risk of osteoporosis and pathological fractures.

## 6. Pleiotropic Effects of Vitamin D

The discovery of vitamin D receptors in most human organs and tissues has laid the foundation for a deeper understanding of the effects of vitamin D on the human body.

### 6.1. Vitamin D and Its Role in Metabolism

Vitamin D receptors have been identified on the surface of the pancreas, liver, muscle, and adipose tissues, determining its role in the regulation of lipid and carbohydrate metabolism [7,39]. Calcitriol regulates insulin synthesis by pancreatic β-cells and maintains tissue sensitivity to insulin. Furthermore, evidence suggests that calcitriol reduces systemic inflammation and helps prevent the development of metabolic syndrome [40,41]. Vitamin D insufficiency and deficiency are considered additional risk factors for the development of type 2 diabetes mellitus, insulin resistance, and metabolic syndrome [40,42].

### 6.2. Vitamin D and Its Role in Reproductive Health

The role of vitamin D in female reproductive health is multifaceted. On one hand, the active form of vitamin D participates in the regulation of sex hormone synthesis and follicular maturation, creating favorable conditions for conception. On the other hand, vitamin D ensures optimal endometrial thickness for embryo implantation [43,44]. Moreover, the placenta and decidual tissue are capable of locally activating calcitriol to support fetal growth and development, as well as immune tolerance, thereby preventing fetal rejection [43]. During pregnancy, adequate 25(OH)D concentrations influence placental development, contribute to the formation of the fetal lungs and the musculoskeletal system, and play a role in immune regulation [43,45]. It has been established that vitamin D plays an equally important role in male reproductive health. According to published data, optimal vitamin D status is associated with key qualitative characteristics of semen, including sperm concentration, motility, and Kruger morphological criteria [46].

### 6.3. Vitamin D and Its Role in Immune Support

Calcitriol enhances the activity of macrophages and neutrophils, increasing their antioxidant capacity and their ability to perform phagocytosis. In the epithelial cells of the respiratory tract, vitamin D contributes to maintaining barrier function. It has been observed that among individuals with normal 25(OH)D concentrations, the incidence of respiratory infections and complicated bacterial respiratory tract infections is significantly lower [47,48,49]. Vitamin D plays a protective role in the development of autoimmune diseases by inhibiting the activation and differentiation of B lymphocytes, preventing their proliferation and differentiation into plasma cells, as well as by stimulating T-helper and regulatory T-cells responsible for anti-inflammatory and immune-tolerant responses. In addition, vitamin D suppresses the expression of pro-inflammatory cytokines. Thus, vitamin D enhances anti-infective defense while simultaneously reducing the risk of chronic inflammation and autoimmune reactions.

### 6.4. Vitamin D and Its Role in Antitumor Protection

Calcitriol exerts antiproliferative effects, regulates cell differentiation, and inhibits neoangiogenesis, thereby reducing the risk of cancer development [50,51].

### 6.5. Vitamin D and Its Role in the Cardiovascular System

Vitamin D is involved in the regulation of the renin–angiotensin–aldosterone system, reducing renin activity and contributing to the normalization of blood pressure [50]. Thus, vitamin D is an important regulator of multiple systems of the human body, including not only calcium and phosphate metabolism but also reproductive function, immunity, the cardiovascular system, and antitumor protection. The pleiotropic effects of vitamin D highlight that its deficiency may have clinical consequences; therefore, the prevention and correction of vitamin D deficiency may play a significant role in the comprehensive therapy and prevention of chronic diseases.

## 7. Diseases Associated with Vitamin D Deficiency and Insufficiency

It is well known that vitamin D insufficiency and deficiency are associated with disturbances in bone and mineral metabolism. A low 25(OH)D leads to decreased absorption of calcium and phosphorus, followed by the development of hyperparathyroidism, which results in rickets in children and osteomalacia in adults [8]. In elderly individuals, vitamin D deficiency is an important factor in the development of osteoporosis and increased risk of fractures [14]. These conditions remain the most well-known manifestations of vitamin D insufficiency and have long been considered its key clinical consequences. Vitamin D deficiency with several non-skeletal outcomes, including cancer, autoimmune diseases, and infertility, is heterogeneous. However, in recent decades, a large number of studies have been published confirming that vitamin D deficiency and insufficiency affect not only the skeletal system [4,5,8]. In endocrinology, low 25(OH)D concentrations are considered as an additional risk factor for the development of insulin resistance, type 2 diabetes mellitus, and obesity [40,42,52]. In children and adolescents, low 25(OH)D concentrations are associated with a higher risk of type 1 diabetes mellitus onset, which is related to disturbances in carbohydrate and lipid metabolism, as well as proinflammatory effects [52,53]. Since vitamin D regulates the functions of macrophages and neutrophils, its deficiency is associated with an increased incidence of respiratory tract infections, including ARVI, influenza, and pneumonia [54]. During the COVID-19 pandemic, low 25(OH)D concentrations were observed in most patients with severe disease and higher mortality rates [48,49]. In addition, due to the influence of vitamin D on the functions of T and B lymphocytes, its deficiency is associated with an increased risk of developing autoimmune diseases such as multiple sclerosis, systemic lupus erythematosus, rheumatoid arthritis, psoriasis, and autoimmune thyroiditis [55,56]. Moreover, the blood concentration of 25(OH)D correlates with higher activity of the pathological process [54]. An increasing body of evidence indicates an association between vitamin D deficiency and oncological diseases. It has been established that vitamin D exhibits antiproliferative and antiangiogenic effects; therefore, its deficiency is associated with a higher risk of developing colorectal cancer, breast cancer, and prostate cancer [48,49]. It has also been noted that in oncology patients, vitamin D deficiency and insufficiency are associated with poorer tolerance to anticancer therapy [51]. Particular attention should be given to the role of vitamin D in gynecology. Its deficiency is associated with a higher prevalence of uterine fibroids. Vitamin D exerts an antiproliferative effect on uterine smooth muscle cells, preventing the development of fibroids [57]. Correction of vitamin D deficiency may help slow the growth of existing fibroid nodes. In young women, vitamin D deficiency is associated with PCOS, which is linked to obesity, insulin resistance, hyperandrogenism, metabolic syndrome, and menstrual disorders [32]. Correction of vitamin D deficiency plays a role in the treatment of PCOS by improving carbohydrate metabolism, increasing insulin sensitivity, and restoring the menstrual cycle. Recent data also indicate that endometriosis is associated with vitamin D deficiency [58]. Women with endometriosis more frequently present with low 25(OH)D concentrations compared to the control group [58]. It is assumed that vitamin D may regulate the course of the disease by suppressing proinflammatory cytokines, activating regulatory T cells, and influencing angiogenesis. In experimental models, calcitriol was shown to reduce the proliferation of endometrial cells and the size of ectopic lesions. Moreover, clinical studies have shown that low vitamin D levels correlate with the severity of pain syndrome. During menopause, low 25(OH)D concentrations exacerbate manifestations of osteoporosis, sarcopenia, and cognitive impairment [11,12]. Evidence suggests that vitamin D supplementation as part of combination therapy in women in the early postmenopausal period improves quality of life [11,12]. The influence of vitamin D on the reproductive health of both women and men underlies a wide range of conditions; for example, vitamin D deficiency in either sex may lead to infertility or unsuccessful attempts at in vitro fertilization (IVF). In women, low 25(OH)D concentrations are associated with pregnancy complications such as preeclampsia, gestational diabetes mellitus, and preterm birth. For instance, when blood 25(OH)D concentration falls below 20 ng/mL, the risk of preterm birth increases by 3.3 times compared to optimal levels [43,59]. The most well-known manifestation of vitamin D deficiency in newborns and young children resulting from maternal deficiency during pregnancy is the development of rickets and impaired bone formation [60]. Moreover, numerous studies have demonstrated that maternal vitamin D deficiency during pregnancy may lead to intrauterine growth retardation and fetal hypotrophy [60,61,62]. Low 25(OH)D concentrations in umbilical cord blood are statistically significantly associated with an increased risk of neonatal sepsis [63]. It has been established that in premature infants, the risk of retinopathy of prematurity correlates with 25(OH)D concentrations during antenatal development [64,65]. According to research findings, the incidence of lower respiratory tract infections in the first months of life is higher among newborns whose mothers had blood 25(OH)D concentrations below 30 ng/mL [66]. Furthermore, prospective observation of these groups of children revealed that they are at increased risk of developing asthma and recurrent bronchitis in early childhood [66]. Finally, vitamin D is increasingly being considered in the context of aging and age-associated diseases. Its deficiency contributes to the progression of sarcopenia, impairs muscle function, and increases the risk of falls in elderly patients [9,13]. Thus, vitamin D deficiency and insufficiency are significant risk factors for a wide range of diseases, emphasizing the importance of timely diagnosis, correction of vitamin D deficiency, and prevention of its insufficiency.

## 8. Risk Factors for the Development of Vitamin D Deficiency

According to epidemiological data, the population of Kazakhstan and most Central Asian countries is geographically classified as a high-risk group for the development of vitamin D deficiency and insufficiency, regardless of other factors [14]. However, identifying specific, most vulnerable categories of individuals may have practical significance (Table 1). Etiologically and pathogenetically, the risk factors for the development of vitamin D deficiency can be divided into three main groups:

The systematization of risk factors and their classification according to three key mechanisms forms the foundation for unified clinical approaches within the region and highlights the need to implement algorithms for diagnosing vitamin D deficiency and preventing its insufficiency in the clinical practice of physicians across various specialties.

## 9. Diagnosis of Vitamin D Deficiency

### 9.1. Patient Groups

Population screening of 25(OH)D concentrations in asymptomatic individuals is not recommended due to the lack of proven clinical benefit and cost-effectiveness [64]. However, measurement of 25(OH)D is recommended for patients belonging to the risk groups described in Section 8 or in the presence of clinical manifestations.

### 9.2. Reference Values

“Vitamin D level” is assessed by measuring the serum concentration of 25-hydroxyvitamin D (25(OH)D), which represents the total concentration of the metabolites 25-hydroksyergocalciferol-25(OH)D_2_ and 25-hydroxycholecalciferol-25(OH)D_3_. There are many methods for determining vitamin D status; however, the gold standard is liquid chromatography–tandem mass spectrometry (LC-MS/MS), which provides high specificity and reproducibility [65]. However, using LC-MS/MS may be challenging in lower-resource and rural settings. Immunochemical methods, such as enzyme-linked immunosorbent assay or chemiluminescent immunoassay, may yield overestimated or underestimated results due to cross-reactivity with other vitamin D metabolites [65]; however these methods and their limitations are well recognized by labs. The concentration of 25(OH)D, determined by the LC-MS/MS method, can be expressed either in nanograms per milliliter (ng/mL) or in nanomoles per liter (nmol/L). The choice of measurement units depends on the country; for example, ng/mL is more commonly used in the USA or Poland, whereas nmol/L is typically used in Kazakhstan, Russia, and Germany [65]. In publications, both units are often provided for reader convenience. A quantity of 1 ng/mL corresponds to 2.5 nmol/L; therefore, a conversion factor of 2.5 is used when converting between these units. Population characteristics, climatic conditions, and differences in food fortification programs account for the absence of globally unified reference ranges for 25(OH)D concentrations. In some guidelines, vitamin D deficiency is defined as 25(OH)D below 20 ng/mL (50 nmol/L), while in other international recommendations, it is defined as 25(OH)D concentrations at or below 30 ng/mL (75 nmol/L) [16,27]. For example, the previous endocrine society (USA) guideline defined deficiency as ≤20 ng/mL, insufficiency as 21–29 ng/mL, and optimal vitamin D status as ≥30 ng/mL [64]. We selected 30 ng/mL as a pragmatic clinical threshold based on expert consensus and existing international recommendations that define 30 ng/mL as an adequate or optimal level. Classifications of vitamin D status from severe deficiency to toxic levels were adopted and are provided in Table 2 [3].

Considering the specific characteristics of the Central Asian region and the absence of national protocols, we propose using the classification shown in Table 2 as the most detailed and clinically applicable. Furthermore, similar geographical latitudes (Poland 49–54° N, Central Asia 42–55° N), seasonal insufficiency of solar insolation, low consumption of seafood in the traditional diet, and the absence of large-scale food fortification programs with vitamin D create comparable conditions for the development of vitamin D deficiency among the population. Therefore, the use of this classification is clinically justified for the countries of Central Asia.

## 10. Correction of Vitamin D Deficiency and Insufficiency

Correction of vitamin D deficiency and insufficiency should be carried out using certified medicinal products, as drug certification is a mandatory requirement for registration in Kazakhstan and confirms proven efficacy, safety, and reproducibility of dosing. Vitamin D deficiency may be corrected with appropriate regulated preparations, including supplements where permitted and quality-assured, under medical supervision. The use of dietary supplements (DS) for the correction of vitamin D deficiency is not recommended, due to the lack of standardized doses and sufficient evidence base [66]. For the prevention of vitamin D insufficiency, the use of dietary supplements is permissible but requires caution due to potential discrepancies between the declared and actual concentration of the active substance. In clinical practice, cholecalciferol (vitamin D_3_) preparations are used for the correction of deficiency and the prevention of insufficiency. After administration, cholecalciferol is converted in the body into calcifediol, and subsequently into the active form—calcitriol. In cases of liver or kidney pathology, other forms of vitamin D, such as calcifediol (25(OH)D_3_) or calcitriol (1,25(OH)_2_D_3_), may be considered to be used; however, their administration requires specific medical indications. The Expert Council recommends a two-stage approach to the correction of vitamin D deficiency:

1. Correction phase—administration of therapeutic doses of cholecalciferol until target serum 25(OH)D concentrations are achieved (≥30 ng/mL or 75 nmol/L).
2. Maintenance phase—transition to prophylactic doses of cholecalciferol to prevent a subsequent decrease in 25(OH)D concentration.

### 10.1. Correction Phase—Therapeutic Doses

Therapeutic doses depend on the patient’s age and the severity of deficiency. Additional conditions influencing the therapeutic dosing of vitamin D are the liver disease, the kidney disease, malabsorption syndromes, obesity or overweight, polypharmacy due to multimorbidity or there is an immediate need to restore 25(OH)D to optimal level. Monitoring of 25(OH)D concentrations is performed to assess the efficacy and safety of therapy (Table 3).

### 10.2. Maintain Phase—Prophylactic Doses

After normalization of 25(OH)D concentrations, the patient is switched to prophylactic dosages, which have a broader range depending on the patient’s risk group (Table 4). When prescribing prophylactic doses of cholecalciferol, monitoring of serum 25(OH)D concentrations is not mandatory and is recommended only for patients with impaired vitamin D metabolism (such as those with obesity, malabsorption, osteoporosis, etc.). If prophylactic therapy is continued long-term, the first monitoring can be performed after 3–6 months.

## 11. Lifestyle Modification

Lifestyle modification is an important component of the management plan for patients with vitamin D deficiency and insufficiency. It enhances the effectiveness of pharmacological correction and reduces the risk of recurrent deficiency after achieving optimal serum 25(OH)D concentrations. In modern living conditions, limited sun exposure, low consumption of foods rich in vitamin D, and impaired absorption are key risk factors for the development of vitamin D deficiency. In this regard, lifestyle modification measures are aimed at ensuring adequate sun exposure, taking into account age, skin type, geographical location, season, and time of day, as well as at improving the bioavailability of vitamin D through diet and body weight control [67,68,69].

### 11.1. Sun Exposure

The synthesis of vitamin D in the skin occurs under the influence of UVB radiation when the sun’s elevation above the horizon is sufficient (>45°), that is, when a person’s shadow is shorter than their height [2,4]. This period is referred to as the “sun exposure window” and depends on geographical latitude, season, and time of day. In Central Asia, the duration of the sun exposure window during the summer period is approximately two hours before and after noon, while in winter it is reduced to about one and a half hours. At the same time, in winter, in cities located at mid-latitudes (such as Almaty, Bishkek, Tashkent) and farther north, the sun often does not reach the required elevation, making cutaneous vitamin D synthesis impossible [28,70]. For effective vitamin D synthesis, not only the sun exposure window but also the surface area of exposed skin is important. Optimal exposure involves 10–25% of body surface area, which corresponds to an uncovered face, arms, and lower legs. According to data from Holick et al., individuals with light skin phototypes (Fitzpatrick I–II) require only 10–15 min of sun exposure at midday in summer with 10–25% of the body surface exposed, whereas individuals with dark skin phototypes (Fitzpatrick V–VI) need longer exposure times [16,17,26,27]. When a smaller area of the skin is exposed (for example, only the face and hands—about 5%, which is typical for women wearing closed clothing for religious reasons), vitamin D synthesis is insufficient even during the summer months [71,72]. Most international organizations provide similar recommendations: the World Health Organization (WHO) reports that adults living at mid-latitudes require 15–30 min of sun exposure during summer several times per week with 10–25% of body surface area exposed [73,74]. The previous Endocrine Society guidelines recommended 10–15 min of exposure 2–3 times per week under the same conditions [64]. It is important to keep in mind that these recommendations depend on geographical latitude and season. To calculate the optimal duration of sun exposure for cutaneous vitamin D synthesis during the summer period in Central Asia, we developed a model based on the recommendations of the Endocrine Society and the geographical and climatic characteristics of the region. Using published data on the variability of the UVB radiation spectrum depending on the season and time of day, a UVB efficiency coefficient was calculated for the region [75,76]. The optimal exposure time for each month was calculated using the following formula:

T month = T reference/UVB coefficient

where T month is the optimal duration of sun exposure for a given month; T reference is the recommended exposure time under optimal conditions (i.e., during the solar window in summer with 10–25% of body surface area exposed); and UVB coefficient is the coefficient of relative UVB radiation efficiency for a specific month and region compared to the summer maximum [75,76]. In Central Asia, the southern part of the region, including Turkmenistan, the southern areas of Uzbekistan and Tajikistan, is located between 35 and 40° N lat., while the majority of the territory, including cities such as Tashkent, Bishkek, and Almaty, lies between 41 and 45° N lat. Only the northern part of Kazakhstan is situated above 46° N lat.; the optimal duration of sun exposure during the summer period, with 10–25% of body surface area exposed, is approximately 20–25 min for the southern and central parts of the region and about 30–40 min for the northern areas. Detailed methodological assumptions, UVB efficiency coefficients, and estimated sun-exposure values for selected Central Asian cities are provided in Supplementary Materials File S1.

### 11.2. Special Populations

Certain population groups require a specific approach. Direct sun exposure is not recommended for infants and toddlers under the fourth year of age due to the risk of burns (and the increased risk of melanoma in the future); therefore, their primary source of vitamin D should come from food and supplements. For children aged 4–10 years, short sun exposures (10–15 min, 2–3 times per week) with the arms and legs uncovered (10–25% of body surface area) are acceptable. In older adults, vitamin D synthesis is reduced by 2–4 times, so sun exposure cannot be considered a reliable source of cutaneous vitamin D synthesis. In the context of the growing beauty and wellness industry, it is important to consider the effect of sunscreens on cutaneous vitamin D synthesis. The use of creams with a sun protection factor (SPF) of ≥15 blocks up to 95% of UVB radiation, sharply reducing vitamin D synthesis even with sufficient skin exposure [77]. At the same time, complete avoidance of SPF is not recommended, given its proven effectiveness in preventing photodamage and skin cancer [78]. The optimal strategy is considered to be short-term skin exposure (10–20 min) without SPF, followed by sunscreen application [78].

### 11.3. Diet

The second most significant source of vitamin D is animal-derived foods rich in this vitamin, such as fatty sea fish (salmon, herring, mackerel, tuna), fish oil and seafood, egg yolk, dairy products, butter, as well as fortified or artificially enriched foods (milk, yogurt, juices, cereals) [3,26]. In most parts of Central Asia, where there is no access to the sea, fish consumption is low, and food fortification with vitamin D is uncommon; therefore, the habitual diet does not meet vitamin D requirements. However, including fatty fish in the diet 2–3 times per week is considered an additional factor in maintaining vitamin D levels, especially during the winter period when its synthesis in the skin is limited [3,6].

### 11.4. Body Weight Control

Excess body weight and obesity are associated with reduced bioavailability of vitamin D, since a significant portion of it is deposited in adipose tissue and enters the bloodstream to a lesser extent. As a result, even with adequate sun exposure, the concentration of 25(OH)D in patients with obesity is markedly and significantly lower than in individuals with a normal body mass index (BMI) [61]. Maintaining a normal body weight, reducing weight in patients with obesity, and preventing its development are considered key lifestyle modification measures that contribute to achieving adequate serum 25(OH)D concentrations.

## 12. Clinical Pharmacology, Safety, and Toxicity of Vitamin D

In the Republic of Kazakhstan, the circulation of medicinal products is regulated by the Code of the Republic of Kazakhstan “On the health of the people and the healthcare system”. According to this Code, medicinal products must comply with GMP (Good Manufacturing Practice) requirements and undergo state registration, as well as evaluation of quality, safety, and efficacy, including monitoring of adverse reactions and undesirable effects; dietary supplements (DS), on the other hand, are regulated as food products under the sanitary regulations of the Republic of Kazakhstan [79]. Their registration does not require confirmation of therapeutic efficacy or the conduct of clinical trials.

In the Republic of Uzbekistan, the circulation of medicinal products is regulated by the national regulatory authority. The registration process requires mandatory compliance with GMP standards, including certification from both the country of manufacture and the local regulatory authority. Applicants must submit comprehensive documentation supporting the evaluation of product quality, safety, and efficacy, including pharmacovigilance data and monitoring of adverse events. Dietary supplements are regulated as food products in accordance with the sanitary legislation of the Republic of Uzbekistan. Their registration requires toxicological assessment and confirmation of product composition, without the comprehensive clinical evaluation required for medicinal products [80,81].

Data from Kyrgyzstan, Tajikistan, and Turkmenistan were inaccessible during manuscript preparation. More population-based studies, standardized 25(OH)D measurement, and country-level surveillance are needed in Central Asia.

### 12.1. Safety of Vitamin D

In patients at risk of vitamin D hypersensitivity, supplementation should be supervised and carried out carefully, in an individual manner, always monitored with serum Ca, serum parathyroid hormone (PTH), serum 25(OH)D, serum 1,25(OH)_2_D and 24 h calciuria (preferred over urinary Ca/creatinine ratio). Patients who suffer from chronic granuloma-forming disorders including sarcoidosis, tuberculosis, and chronic fungal infections and some patients with lymphoma have activated macrophages that produce 1,25(OH)_2_D in an unregulated fashion. These patients may require vitamin D treatment to raise their serum 25(OH)D to approximately 25 ng/mL [6,7,26,27]. The 25(OH)D concentrations should be monitored, because hypercalciuria and hypercalcemia are usually observed when the 25(OH)D is above 30 ng/mL. Patients with primary hyperparathyroidism and hypercalcemia are often vitamin D deficient. It is important to correct their vitamin D deficiency and maintain sufficiency, i.e., 25(OH)D > 30 ng/mL; however, supplementation with cholecalciferol should be cautious to prevent further increases in the serum or urinary calcium concentration [6,7]. Patients with chronic kidney disease, especially dialysis patients, kidney transplant recipients, are at the risk of inadequate activation of vitamin D by hydroxylation in position 1α by CYP27B1 and deactivation by CYP24A1, because both enzymes are mostly active in proximal tubules of the kidneys. SLC34A1 gene mutation, CYP24A1 gene mutation, hypercalciuria, hypercalcemia, nephrolithiasis, nephrocalcinosis, or history of other types of vitamin D hypersensitivity in an individual or family members should be considered before starting vitamin D supplementation [6,7,26,27].

### 12.2. Safe Therapeutic Dose

When prescribing cholecalciferol for therapeutic purposes to correct vitamin D deficiency, one should rely on the upper permissible dosage levels, which for in-term born infants are 2000 IU/day, for toddlers and young children 1–10 years are 4000 IU/day, and for adolescents and adults (including pregnant and lactating women) the therapeutic doses start from 4000 IU/day up to 10,000 IU/day, especially for adults with obesity or malabsoption syndromes, liver or kidney disease, etc. [3,6]. It should be emphasized that treatment of vitamin D deficiency requires higher doses than doses recommended for prophylaxis. Special attention should be given to the treatment of neonates and infants where we suggested the use of 2000 IU/d as a treatment dose for vitamin D deficiency, including monitoring of 25(OH)D no later than 4–6 weeks after the start of therapy. The use of therapeutic doses was evaluated in an RCT in a group of 40 children aged 8–24 months with vitamin D deficiency, i.e., 25(OH)D < 20 ng/mL. The safety and effectiveness of doses of 2000 IU/d of vitamin D_2_, 50,000 IU/week of vitamin D_3_, and 2000 IU/d of vitamin D_3_ were compared over a 6-week period. In the group treated with 2000 IU/d of vitamin D_2_, after 6 weeks of the study, 25(OH)D concentration increased from 15.7 ng/mL at baseline to 43.9 ng/mL at the end of study. In the group treated with 50,000 IU/week (equivalent of 7143 IU per day), an increase from 13.8 ng/mL to 44.0 ng/mL was observed. Finally, in the 2000 IU/d group treated for 6 weeks, the 25(OH)D concentration increased from 13.7 ng/mL to 41.2 ng/mL, without any side effects in all groups subjected to this RCT [82]. In the paper titled “Global consensus recommendations on prevention and management of nutritional rickets,” Muns et al. recommended the dose of vitamin D of minimum 2000 IU/d for 3 months for infants aged < 12 months with vitamin D deficiency [83].

According to the instructions officially approved by the regulatory authorities in the Central Asian countries—Kazakhstan, Kyrgyzstan, Uzbekistan—the intermittent doses in cases of vitamin D deficiency are also higher, for example 50,000 IU once weekly (equivalent of 7143 IU per day) for 8 weeks and in cases of insufficiency, 50,000 IU given once weekly for 4 weeks. The same issue relates to doses of 20,000 IU (equivalent of 2857 IU/day) and 30,000 IU (equivalent of 4286 IU/day). These values are established based on international recommendations and confirmed by the decision of the Expert Council [3,6,84].

### 12.3. Safe Prophylactic Dose

Prophylactic doses of vitamin D have a high safety profile and can be used long-term without the risk of developing toxic effects, as well as without the need for laboratory monitoring in healthy individuals. According to international recommendations as well as our resolutions, prophylactic doses for in-term born neonates and young infants aged 0–6 months are 400–500 IU/day (10 µg/day) of cholecalciferol from first days of life, regardless of the feeding method; for infants aged 6–12 months the recommended dose of cholecalciferol is 400–600 IU/day (10–15 µg/day), depending on the daily amount of vitamin D consumed with meals. In healthy children aged 1–3 years, supplementation should be based on cholecalciferol administration provided in a daily dose of 500–600 IU (15 g/day) and, due to age-related restrictions of sunbathing, is recommended throughout the year. In healthy children aged 4–10 years sunbathing with uncovered forearms and legs for 15–30 min between 10 a.m. and 3 p.m. without sunscreen, starting from May until the end of September, cholecalciferol supplementation is not necessary, although still recommended and safe. If these guidelines are not fulfilled in healthy children aged 4–10 years, supplementation of cholecalciferol in a dose of 600–1000 IU/day (15–25 µg/day) is recommended throughout the year, based on body weight and the dietary vitamin D intake. In healthy adolescents and adults sunbathing with uncovered forearms and legs for 30–45 min between 10 a.m. and 3 p.m. without sunscreen, starting from May until the end of September, cholecalciferol supplementation is not necessary, although still recommended and safe. If these guidelines are not fulfilled, supplementation based on cholecalciferol in a dose of 1000–2000 IU/day (25–50 µg/day) is recommended throughout the year, based on body weight and the dietary vitamin D intake. For pregnant women, the recommended daily dose is 2000 IU. For elderly individuals, cholecalciferol supplementation with doses 2000–4000 IU/day is recommended for prophylactics of vitamin D deficiency/insufficiency [3,6]. These values have been confirmed and approved by the members of our Expert Council who participated in the preparation of this material.

### 12.4. Toxicity and Overdose Risks

The established threshold of possible vitamin D toxicity in the blood is 25(OH)D > 100 ng/mL (250 nmol/L) [3]. Chronic elevation of vitamin D levels in the blood may lead to symptoms of hypervitaminosis D, such as anorexia, nausea, polyuria, arrhythmias, muscle weakness, hypercalcemia, and impaired renal function. In severe cases, hypercalcemia may lead to vascular and soft tissue calcification and damage to organs, including the heart and kidneys. In the event of hypervitaminosis D symptoms, a comprehensive approach is required: immediate discontinuation of cholecalciferol preparations and restriction of calcium intake from food and supplements. The patient should be provided with adequate oral and intravenous hydration to increase calcium excretion in the urine. In severe cases, medical management is required, including the use of glucocorticosteroids to reduce calcium absorption in the intestine, loop diuretics to enhance renal calcium excretion, and bisphosphonates to inhibit bone resorption. Patient monitoring should include regular assessment of calcium, creatinine, glomerular filtration rate (GFR), and 25(OH)D levels in the blood.

## 13. Conclusions

As a result of the meeting of the Expert Council “Optimization of the strategy for the prevention of vitamin d deficiency: from scientific data to real practice in Central Asia,” which included specialists in endocrinology, obstetrics and gynecology, rheumatology, clinical pharmacology, and family medicine, a unified interdisciplinary resolution was formed, reflecting the approaches to the diagnosis, correction of vitamin D deficiency, and prevention of its insufficiency in the countries of Central Asia.

The widespread problem of vitamin D deficiency across various social groups is caused by geographical, climatic, cultural, and social factors. Considering the absence of national protocols and strategies, the development of unified recommendations is a top priority for public health professionals. The key value of this resolution is its regional and implementation-oriented focus. Although vitamin D deficiency has been widely discussed in international literature, Central Asia remains underrepresented in consensus documents and clinical recommendations. The present resolution addresses this gap by adapting the available scientific evidence and expert experience to the geographical, climatic, nutritional, cultural, and healthcare realities of the region.

The Expert Council considers this classification to be clinically applicable for Central Asia due to comparable geographical and seasonal conditions, limited dietary vitamin D intake, and the absence of large-scale fortification programs. At the same time, the Council acknowledges that international cut-off points for vitamin D status differ between guidelines. Therefore, the target level of ≥30 ng/mL/75 nmol/L should be interpreted as a pragmatic clinical target for prevention and correction strategies, particularly in risk groups, rather than as an absolute universal threshold for all populations and outcomes.

The Expert Council recommends that correction of confirmed vitamin D deficiency should be performed using certified medicinal products that have undergone state registration and quality evaluation. Correction should follow a two-stage approach: first, a correction phase aimed at achieving target serum 25(OH)D concentrations; second, a maintenance phase using prophylactic doses to prevent recurrent insufficiency or deficiency. Therapeutic dosing should be distinguished from routine prophylactic supplementation and should be accompanied by appropriate clinical and laboratory monitoring, especially in children, pregnant women, patients with obesity, malabsorption, osteoporosis, chronic kidney or liver disease, granulomatous diseases, hypercalcemia, nephrolithiasis, sarcoidosis, malignancies associated with hypercalcemia, or other disorders affecting calcium-phosphate and vitamin D metabolism.

Prevention of vitamin D insufficiency should be comprehensive and should include pharmacological support with cholecalciferol in recommended prophylactic doses, lifestyle modification, adequate and safe sun exposure during appropriate seasons, increased consumption of dietary sources of vitamin D, and body weight control. In generally healthy individuals receiving age-appropriate prophylactic doses, routine laboratory monitoring may not always be necessary. However, monitoring is recommended for patients with impaired vitamin D metabolism, high-risk clinical conditions, long-term high-dose therapy, or suspected toxicity.

The proposed recommendations should be regarded as a practical regional framework for clinical and public health decision-making. They may serve as a foundation for the development of national clinical protocols, physician education programs, prevention strategies, and further interdisciplinary collaboration in Central Asia. At the same time, implementation should take into account differences in national regulatory systems, availability of laboratory testing, access to certified vitamin D preparations, healthcare resources, and the feasibility of large-scale screening or supplementation programs.

Further work should include additional population-based epidemiological studies in Central Asian countries, standardization of 25(OH)D measurement approaches, evaluation of the feasibility and cost-effectiveness of preventive strategies, expansion of country-specific regulatory analysis, and regular updates of the consensus as new regional and international evidence becomes available.

Coordination between the professional medical community, health authorities, regulatory bodies, and public health institutions is essential for improving awareness, strengthening prevention, and reducing the burden of vitamin D deficiency and insufficiency in Central Asia.

## Figures and Tables

**Table 1:** Risk groups for the development of vitamin D deficiency.

Groups with Reduced Synthesis or Limited Intake of Vitamin D	Groups with Increased Physiological Need for Vitamin D	Groups with Impaired Vitamin D Metabolism
**Tender-age infants** Direct sun exposure is contraindicated Limited dietary intake	**Infants, toddlers and adolescents** Rapid growth and mineralization of long bones require additional vitamin D	**Individuals with obesity** Bioavailability of vitamin D is reduced due to its sequestration in adipose tissue and systemic dilution in increased body size
**Individuals over 60 years of age** Cutaneous synthesis of vitamin D is reduced by 2–4 times	**Pregnant women** High demand to ensure adequate vitamin D supply for both the mother and the fetus	**Patients with chronic gastrointestinal, liver, or kidney diseases** Absorption and metabolism of vitamin D are impaired
**Office workers and residents of large cities** Sun exposure is significantly limited Air pollution reduces the penetration of UVB rays Use of sun protection factors (SPF)	**Breastfeeding women** Additional consumption of vitamin D due to lactation	**Patients with oncological diseases** Vitamin D consumption decreases Anticancer therapy may disrupt vitamin D metabolism
**Individuals with limited sun exposure due to social and cultural factors** Wearing closed clothing for religious reasons Individuals with limited mobility		**Patients with chronic autoimmune diseases** **(rheumatoid arthritis, systemic lupus erythematosus, multiple sclerosis, thyroiditis, type 1 diabetes mellitus, Crohn’s disease, celiac disease, vitiligo)** Systemic inflammation disrupts vitamin D metabolism Low 25(OH)D concentration is related to increased release of pro-inflammatory cytokines
**Individuals with dark skin phototype** Darker skin contains more melanin, which blocks UVB rays and reduces vitamin D synthesis		**Individuals taking medications that reduce absorption or activation of vitamin D** hypolipidemic, antiepileptic, glucocorticosteroids, immunosuppressants, antifungal agents, anti-obesity drugs
**Vegetarians/Vegans** Exclusion of foods that are natural sources or support the maintenance of vitamin D status		

**Table 2:** Reference categories of 25(OH)D concentrations.

Category	ng/mL	nmol/L
Severe deficiency	<10	<25
Deficiency	10–20	25–50
Insufficiency	20–30	50–75
Optimal level	30–50	75–125
High level	50–100	125–250
Possibility of toxic level	>100	>250
Toxicity level	>250	>625

**Table 3:** Therapeutic doses of cholecalciferol.

Age	Dosage	Monitoring
From birth to 12 months of age	2000 IU/day *	serum 25(OH)D concentration control assay no later than 4–6 weeks after
Age 1–10 years	4000 IU/day	serum 25(OH)D concentration control assay no later than 6–8 weeks after
Age 11–18 years	4000 IU/day or 7000 IU/week or 10,000 IU/week or 20,000 IU taken biweekly or 30,000 IU taken biweekly or 30,000 IU/month or 50,000 IU/month	serum 25(OH)D concentration control assay considered 8–12 weeks after, but not later than up to 3 months after, depending on a dose of therapy
Adults (over 19 years)	4000 IU/day or 7000 IU/week or 10,000 IU/week or 20,000 IU taken biweekly or 30,000 IU taken biweekly or 30,000 IU/month or 50,000 IU/month; In some conditions the intermittent doses of 7000 IU, or 10,000 IU can be administrated in daily dosing, and 20,000 IU, or 30,000 IU, or 50,000 IU in weekly dosing	serum 25(OH)D concentration control assay considered 8–12 weeks after, but not later than up to 3 months after, depending on a dose of therapy

* 2000 IU/day in infants younger than 12 months refers to treatment of confirmed deficiency, not routine prevention.

**Table 4:** Prophylactic doses of cholecalciferol.

Age/Group	Dosage
Neonates and infants (0–6 months)	400–500 IU/day (10 µg/day)
Infants (6–12 months)	400–600 IU/day (10–15 µg/day)
Young Children (1–3 years)	500–600 IU (15 g/day)
Older Children (4–10 years)	600–1000 IU/day (15–25 µg/day)
Adolescents (11–18 years)	1000–2000 IU/day
Adults (19–65 years)	1000–2000 IU/day
Elderly (>65 years)	2000–4000 IU/day
Pregnant and breastfeeding women	2000 IU/day
Patients with obesity (BMI ≥ 30 kg/m^2^)	Double dose compared to peers without obesity

## Data Availability

No new data were created or analyzed in this study.

## Supplementary Materials

The following supporting information can be downloaded at: https://www.mdpi.com/article/10.3390/nu18132122/s1, File S1. Methodological Details of the Sun-Exposure Model for Cutaneous Vitamin D Synthesis in Central Asia; Table S1. Relative monthly UVB efficiency coefficients used in the model; Table S2. Estimated duration of midday sun exposure required for cutaneous vitamin D synthesis by latitude zone; Table S3. Approximate sun-exposure estimates for selected Central Asian cities.

## List of Abbreviations

1,25(OH)_2_D_3_	calcitriol, 1,25-dihydroxyvitamin D_3_
25(OH)D	calcidiol, total 25-hydroxyvitamin D
25(OH)D_3_	calcifediol, 25-hydroxyvitmin D3
ARVI	acute respiratory viral infections
BMI	body mass index
CI	confidence interval
COVID-19	coronavirus disease 2019
DS	dietary supplements
Full name	family name, given name, patronymic
GFR	glomerular filtration rate
GMP	Good Manufacturing Practice
IU/day	international units per day
IU/month	international units per month
IU/week	international units per week
IVF	in vitro fertilization
LC-MS/MS	liquid chromatography–tandem mass spectrometry
N lat.	northern latitude
ng/mL	nanograms per milliliter
nmol/L	nanomoles per liter
PCOS	polycystic ovary syndrome
SPF	sun protection factor
UVB	ultraviolet radiation of spectrum B
Vitamin D_2_	ergocalciferol
Vitamin D_3_	cholecalciferol
WHO	World Health Organization

## References

[1] Van Schoor N.M., Lips P. (2011). Worldwide vitamin D status. Best Pract. Res. Clin. Endocrinol. Metab..

[2] Bikle D.D. (2000). Vitamin D: Production, metabolism, and mechanism of action. Endotext [Internet].

[3] Pludowski P., Takacs I., Boyanov M., Belaya Z., Diaconu C.C., Mokhort T., Zherdova N., Rasa I., Payer J., Pilz S. (2022). Clinical practice in the prevention, diagnosis and treatment of vitamin D deficiency: A central and eastern European expert consensus statement. Nutrients.

[4] Janoušek J., Pilařová V., Macáková K., Nomura A., Veiga-Matos J., da Silva D.D., Remião F., Saso L., Malá-Ládová K., Malý J. (2022). Vitamin D: Sources, physiological role, biokinetics, deficiency, therapeutic use, toxicity, and overview of analytical methods for detection of vitamin D and its metabolites. Crit. Rev. Clin. Lab. Sci..

[5] Demay M.B., Pittas A.G., Bikle D.D., Diab D.L., Kiely M.E., Lazaretti-Castro M., Lips P., Mitchell D.M., Murad M.H., Powers S. (2024). Vitamin D for the prevention of disease: An endocrine society clinical practice guideline. J. Clin. Endocrinol. Metab..

[6] Płudowski P., Kos-Kudła B., Walczak M., Fal A., Zozulińska-Ziółkiewicz D., Sieroszewski P., Peregud-Pogorzelski J., Lauterbach R., Targowski T., Lewiński A. (2023). Guidelines for preventing and treating vitamin D deficiency: A 2023 update in Poland. Nutrients.

[7] Liberman U., Bikle D.D. (2000). Disorders in the action of vitamin D. Endotext [Internet].

[8] Bouillon R., Marcocci C., Carmeliet G., Bikle D., White J.H., Dawson-Hughes B., Lips P., Munns C.F., Lazaretti-Castro M., Giustina A. (2019). Skeletal and extraskeletal actions of vitamin D: Current evidence and outstanding questions. Endocr. Rev..

[9] Verlaan S., Maier A.B., Bauer J.M., Bautmans I., Brandt K., Donini L.M., Maggio M., McMurdo M.E., Mets T., Seal C. (2018). Sufficient levels of 25-hydroxyvitamin D and protein intake required to increase muscle mass in sarcopenic older adults—The PROVIDE study. Clin. Nutr..

[10] Saraf R., Morton S.M.B., Camargo C.A., Grant C.C. (2016). Global summary of maternal and newborn vitamin D status—A systematic review. Matern. Child Nutr..

[11] Purdue-Smithe A.C., Whitcomb B.W., Szegda K.L., Boutot M.E., Manson J.E., Hankinson S.E., Rosner B.A., Troy L.M., Michels K.B., Bertone-Johnson E.R. (2017). Vitamin D and calcium intake and risk of early menopause. Am. J. Clin. Nutr..

[12] Alinia T., Sabour S., Hashemipour M., Hovsepian S., Pour H.R., Jahanfar S. (2023). Relationship between vitamin D levels and age of menopause and reproductive lifespan: Analysis based on the National Health and Nutrition Examination Survey (NHANES) 2001–2018. Eur. J. Obstet. Gynecol. Reprod. Biol..

[13] Hirani V., Cumming R.G., Naganathan V., Blyth F., Le Couteur D.G., Hsu B., Handelsman D.J., Waite L.M., Seibel M.J. (2018). Longitudinal associations between vitamin D metabolites and sarcopenia in older Australian men: The concord health and aging in men project. J. Gerontol. Ser. A.

[14] Thanapluetiwong S., Chewcharat A., Takkavatakarn K., Praditpornsilpa K., Eiam-Ong S., Susantitaphong P. (2020). Vitamin D supplement on prevention of fall and fracture. Medicine.

[15] Gromova O., Doschanova A., Lokshin V., Tuletova A., Grebennikova G., Daniyarova L., Kaishibayeva G., Nurpeissov T., Khan V., Semenova Y. (2020). Vitamin D deficiency in Kazakhstan: Cross-sectional study. J. Steroid Biochem. Mol. Biol..

[16] Holick M.F. (2007). Vitamin D Deficiency. N. Engl. J. Med..

[17] Webb A.R., Holick M.F. (1988). The role of sunlight in the cutaneous production of vitamin D3. Annu. Rev. Nutr..

[18] Hinote B.P., Cockerham W.C., Abbott P. (2009). Psychological distress and dietary patterns in eight post-Soviet republics. Appetite.

[19] Shovaliev I.K., Boĭkulov A.A. (2002). Biological value of the average daily diets of elderly and old individuals in Uzbekistan. Gig. Sanit..

[20] Barth-Jaeggi T., Zandberg L., Bahruddinov M., Kiefer S., Rahmarulloev S., Wyss K. (2020). Nutritional status of Tajik children and women: Transition towards a double burden of malnutrition. Matern. Child Nutr..

[21] Bromage S., Tazhibayev S., Zhou X., Liu C., Tserenkhuu E., Dolmatova O., Khishignemekh M., Musurepova L., Tsolmon S., Tsendjav E. (2025). Longitudinal analysis of lifestyle risk factors, nutrition status and drivers of food choice among urban migrants in Ulaanbaatar, Mongolia, and Almaty, Kazakhstan: A formative study. Public Health Nutr..

[22] Akhmetova V., Balji Y., Kandalina Y., Iskineyeva A., Mukhamejanova A., Baspakova A., Uzakov Y., Issayeva K., Zamaratskaia G. (2022). Self-reported consumption frequency of meat and fish products among young adults in Kazakhstan. Nutr. Health.

[23] Golastani B., Poursafa P., Zarean M., Yazdi M., Heidari-Beni M., Kelishadi R. (2024). Relationship between air pollution and serum vitamin D levels: A systematic review and meta-analysis. Adv. Biomed. Res..

[24] Richter J., Větvička V., Král V., Šíma P., Stiborová I., Richterová S., Dobiášová L.R. (2023). Low levels of vitamin D in population exposed to significant environmental pollution. Cas. Lek. Ceskych.

[25] Mousavi S.E., Amini H., Heydarpour P., Chermahini F.A., Godderis L. (2019). Air pollution, environmental chemicals, and smoking may trigger vitamin D deficiency: Evidence and potential mechanisms. Environ. Int..

[26] Holick M.F., Chen T.C. (2008). Vitamin D deficiency: A worldwide problem with health consequences. Am. J. Clin. Nutr..

[27] Holick M.F. (2017). The vitamin D deficiency pandemic: Approaches for diagnosis, treatment and prevention. Rev. Endocr. Metab. Disord..

[28] Karibayeva I., Bilibayeva G., Yerzhanova A., Alekesheva R., Iglikova A., Maxudova M., Ussebayeva N. (2024). Prevalence of vitamin D deficiency among adults in kazakhstan: A systematic review and meta-analysis. Medicina.

[29] Yerezhepov D., Gabdulkayum A., Akhmetova A., Kozhamkulov U.A., Rakhimova S.E., Kairov U.Y., Zhunussova G., Kalendar R.N., Akilzhanova A. (2024). Vitamin D status, VDR, and TLR polymorphisms and pulmonary tuberculosis epidemiology in kazakhstan. Nutrients.

[30] Izmailovich M., Gazaliyeva M., Glushkova N., Semenova Y., Burankulova S., Shaydarova S., Bayazitova M., Kuznetsova I. (2022). Association between serum 25-hydroxyvitamin D concentration and severity of seasonal allergic rhinitis in Karaganda region (Kazakhstan). J. Clin. Med. Kazakhstan.

[31] Zhumina A.G., Li K., Konovalova A.A., Li Y.A., Ishmuratova M.Y., Pogossyan G.P., Danilenko M. (2020). Plasma 25-Hydroxyvitamin D levels and VDR gene expression in peripheral blood mononuclear cells of leukemia patients and healthy subjects in central Kazakhstan. Nutrients.

[32] Safi A., Orazov M., Kalinchenko S. (2020). The role of cholecalciferol deficiency in the pathogenesis of polycystic ovary syndrome. Women’s Health.

[33] Algazina T., Touir G., Pshembayeva S., Jetpisbayeva Z., Batpenova G. (2019). The role of vitamin D in the development of psoriasis and acne. Georgian Med. News.

[34] Nugmanova Z., Patel N., Akhmetova G.M., Kurmangalieva G.S., Abdumananova M.K., Akanov A.A., Kovtunenko N.G., McNutt L.-A. (2015). Relationship between vitamin D and human immunodeficiency virus viral load among HIV-infected patients in Kazakhstan. J. Infect. Dev. Ctries..

[35] Buyuklyanov A.I., Atambayeva R.M., Esenamanova M.K., Kochkorova F.A. (2024). Kyrgyz Respublikasynyn Kalkynyn D Vitamini Menen Kamsy`z Bolushuna Baa Beruyu [Assessment of vitamin D supply in the population of the Kyrgyz Republic]. Euroasian Health J..

[36] Akhmedova D.I., Kamilova A.T., Shamsiev F.M. (2019). Uroven’ vitamina D u detei rannego vozrasta v nekotorykh regionakh Respubliki Uzbekistan [Vitamin D levels in young children in selected regions of the Republic of Uzbekistan]. Pediatriya.

[37] Chernysh P.P. (2019). Obespechennost’ vitaminom D naseleniya razlichnykh vozrastnykh grupp, prozhivayushchikh v gorode Tash-kente [Vitamin D status in different age groups of the population residing in the city of Tashkent]. Zhurnal Teor. Klin. Meditsiny J. Theor. Clin. Med..

[38] Irnazarova D.H., Yuldasheva D.Y., Najmutdinova D.K., Kayumova D.T., Sadikova D.R., Atahodjayeva F.A., Ahmedova G. (2020). Vitamin D status in women with uterine fibroids (UF) of the Uzbek population. J. Crit. Rev..

[39] Giustina A., Bilezikian J.P., Adler R.A., Banfi G., Bikle D.D., Binkley N.C., Bollerslev J., Bouillon R., Brandi M.L., Casanueva F.F. (2024). Consensus statement on vitamin D status assessment and supplementation: Whys, whens, and hows. Endocr. Rev..

[40] Strange R.C., E Shipman K., Ramachandran S. (2015). Metabolic syndrome: A review of the role of vitamin D in mediating susceptibility and outcome. World J. Diabetes.

[41] Saitov A.R., Biek A.Y., Dobrynina I.Y., Kovalenko T.N., Aryamkina O.L. (2021). Defitsit vitamina D pri metabolicheskom sindrome i nealkogol’noi zhirovoi bolezni pecheni [Vitamin D deficiency in metabolic syndrome and non-alcoholic fatty liver disease]. Vestn. SurGU. Meditsina.

[42] Sacerdote A., Dave P., Lokshin V., Bahtiyar G. (2019). Type 2 diabetes mellitus, insulin resistance, and vitamin D. Curr. Diabetes Rep..

[43] Kiely M.E., Wagner C.L., Roth D.E. (2020). Vitamin D in pregnancy: Where we are and where we should go. J. Steroid Biochem. Mol. Biol..

[44] Garbedian K., Boggild M., Moody J., Liu K.E. (2013). Effect of vitamin D status on clinical pregnancy rates following in vitro fertilization. CMAJ Open.

[45] Mailhot G., White J.H. (2020). Vitamin D and Immunity in Infants and Children. Nutrients.

[46] Ciccone I.M., Costa E.M., Pariz J.R., Teixeira T.A., Drevet J.R., Gharagozloo P., Aitken R.J., Hallak J. (2021). Serum vitamin D content is associated with semen parameters and serum testosterone levels in men. Asian J. Androl..

[47] Martineau A.R., Jolliffe D.A., Hooper R.L., Greenberg L., Aloia J.F., Bergman P., Dubnov-Raz G., Esposito S., Ganmaa D., Ginde A.A. (2017). Vitamin D supplementation to prevent acute respiratory tract infections: Systematic review and meta-analysis of individual participant data. BMJ.

[48] Bikle D.D. (2023). Vitamin D and long COVID: Is there a role in prevention or treatment?. J. Clin. Endocrinol. Metab..

[49] Goddek S. (2020). Vitamin D3 and K2 and their potential contribution to reducing the COVID-19 mortality rate. Int. J. Infect. Dis..

[50] Manson J.E., Cook N.R., Lee I.M., Christen W., Bassuk S.S., Mora S., Gibson H., Gordon D., Copeland T., D’Agostino D. (2019). Vitamin D supplements and prevention of cancer and cardiovascular disease. N. Engl. J. Med..

[51] Muñoz A., Grant W.B. (2022). Vitamin D and cancer: An historical overview of the epidemiology and mechanisms. Nutrients.

[52] Stepan M.D., Vintilescu Ș.B., Streață I., Podeanu M.A., Florescu D.N. (2023). The role of vitamin D in obese children with non-alcoholic fatty liver disease and associated metabolic syndrome. Nutrients.

[53] Klimov L.Y., Zakharova I.N., Kurianinova V.A., Nikitina I.L., Karonova T.L., Malyavskaya S.I., Dolbina S.V., Kasyanova A.N., Ivanova A.V., Atanesyan R.A. (2017). Nedostatochnost’ vitamina D i ozhirenie u detei i podrostkov: Naskol’ko vzaimosvyazany dve global’nye pandemii—Rol’ vitamina D v patogeneze ozhireniya i insulinorezistentnosti (chast’ 1) [Vitamin D deficiency and obesity in children and adolescents: How interrelated are the two global pandemics—The role of vitamin D in the pathogenesis of obesity and insulin resistance (part 1)]. Med. Counc..

[54] Bikle D.D. (2022). Vitamin D Regulation of immune function. Curr. Osteoporos. Rep..

[55] Durá-Travé T., Gallinas-Victoriano F. (2024). Autoimmune thyroiditis and vitamin D. Int. J. Mol. Sci..

[56] Athanassiou L., Kostoglou-Athanassiou I., Koutsilieris M., Shoenfeld Y. (2023). Vitamin D and autoimmune rheumatic diseases. Biomolecules.

[57] Simpson S., Pal L. (2023). Vitamin D and infertility. Curr. Opin. Obstet. Gynecol..

[58] Sayegh L., Fuleihan G.E.-H., Nassar A.H. (2014). Vitamin D in endometriosis: A causative or confounding factor?. Metabolism.

[59] Bodnar L.M., Simhan H.N., Catov J.M., Roberts J.M., Platt R.W., Diesel J.C., Klebanoff M.A. (2014). Maternal vitamin D status and the risk of mild and severe preeclampsia. Epidemiology.

[60] Eckhardt C.L., Gernand A.D., Roth D.E., Bodnar L.M. (2015). Maternal vitamin D status and infant anthropometry in a US multi-centre cohort study. Ann. Hum. Biol..

[61] Santamaria C., Bi W.G., Leduc L., Tabatabaei N., Jantchou P., Luo Z.-C., Audibert F., Nuyt A.M., Wei S.Q. (2018). Prenatal vitamin D status and offspring’s growth, adiposity and metabolic health: A systematic review and meta-analysis. Br. J. Nutr..

[62] Francis E.C., Hinkle S.N., Song Y., Rawal S., Donnelly S.R., Zhu Y., Chen L., Zhang C. (2018). Longitudinal maternal vitamin D status during pregnancy is associated with neonatal anthropometric measures. Nutrients.

[63] Zisi D., Challa A., Makis A. (2019). The association between vitamin D status and infectious diseases of the respiratory system in infancy and childhood. Hormones.

[64] Kabataş E.U., Dinlen N.F., Zenciroğlu A., Dilli D., Beken S., Okumuş N. (2017). Relationship between serum 25-hydroxy vitamin D levels and retinopathy of prematurity. Scott. Med. J..

[65] Jamali N., Wang S., Darjatmoko S.R., Sorenson C.M., Sheibani N. (2017). Vitamin D receptor expression is essential during retinal vascular development and attenuation of neovascularization by 1, 25(OH)2D3. PLoS ONE.

[66] Weiss S.T., Mirzakhani H., Carey V.J., O’cOnnor G.T., Zeiger R.S., Bacharier L.B., Stokes J., Litonjua A.A. (2024). Prenatal vitamin D supplementation to prevent childhood asthma: 15-year results from the Vitamin D Antenatal Asthma Reduction Trial (VDAART). J. Allergy Clin. Immunol..

[67] Holick M.F., Binkley N.C., Bischoff-Ferrari H.A., Gordon C.M., Hanley D.A., Heaney R.P., Murad M.H., Weaver C.M. (2011). Evaluation, treatment, and prevention of vitamin D deficiency: An endocrine society clinical practice guideline. Med. J. Clin. Endocrinol. Metab..

[68] Alexandridou A., Schorr P., Stokes C.S., Volmer D.A. (2023). Analysis of vitamin D metabolic markers by mass spectrometry: Recent progress regarding the “gold standard” method and integration into clinical practice. Mass Spectrom. Rev..

[69] Zemp J., Erol C., Kaiser E., Aubert C.E., Rodondi N., Moutzouri E. (2025). A systematic review of evidence-based clinical guidelines for vitamin D screening and supplementation over the last decade. Arch. Public Health.

[70] Karibayeva I., Bilibayeva G., Iglikova A., Yerzhanova A., Alekesheva R., Maxudova M., Ussebayeva N. (2025). Vitamin D deficiency in Kazakhstani children: Insights from a systematic review and meta-analysis. Medicina.

[71] Religi A., Backes C., Chatelan A., Bulliard J.-L., Vuilleumier L., Moccozet L., Bochud M., Vernez D. (2019). Estimation of exposure durations for vitamin D production and sunburn risk in Switzerland. J. Expo. Sci. Environ. Epidemiol..

[72] Alshamsi M.A., Fatima W., Al Teneiji M.T., Srinivasamurthy S.K. (2025). Vitamin D status among apparently healthy individuals in the UAE: A systematic review. Front. Nutr..

[73] Kift R.C., Webb A.R. (2024). Globally estimated UVB exposure times required to maintain sufficiency in vitamin D levels. Nutrients.

[74] Raymond-Lezman J.R., Riskin S.I. (2023). Benefits and risks of sun exposure to maintain adequate vitamin D levels. Cureus.

[75] Khanna T., Shraim R., Zarkovic M., van Weele M., van Geffen J., Zgaga L. (2022). Comprehensive analysis of seasonal and geographical variation in UVB radiation relevant for vitamin D production in Europe. Nutrients.

[76] Krzyścin J.W., Jarosławski J., Sobolewski P.S. (2011). A mathematical model for seasonal variability of vitamin D due to solar radiation. J. Photochem. Photobiol. B.

[77] Passeron T., Bouillon R., Callender V., Cestari T., Diepgen T., Green A.C., van der Pols J.C., Bernard B.A., Ly F., Bernerd F. (2019). Sunscreen photoprotection and vitamin D status. Br. J. Dermatol..

[78] Young A.R., Narbutt J., Harrison G.I., Lawrence K.P., Bell M., O’Connor C., Olsen P., Grys K., Baczynska K.A., Rogowski-Tylman M. (2019). Optimal sunscreen use, during a sun holiday with a very high ultraviolet index, allows vitamin D synthesis without sunburn. Br. J. Dermatol..

[79] (2020). Code of the Republic of Kazakhstan “On the Health of the People and the Healthcare System”. https://adilet.zan.kz/rus/docs/V2200027946.

[80] Resolution No. 738 of the Cabinet of Ministers of the Republic of Uzbekistan (24 November 2025). https://lex.uz/en/docs/7861699.

[81] Order No. 131 (30 April 2016) issued by the Ministry of Health of the Republic of Uzbekistan. https://cis-legislation.com/document.fwx?rgn=85146.

[82] Gordon C.M., Williams A.L., Feldman H.A., May J., Sinclair L., Vasquez A., Cox J.E. (2008). Treatment of hypovitaminosis D in infants and toddlers. J. Clin. Endocrinol. Metab..

[83] Munns C.F., Shaw N., Kiely M., Specker B.L., Thacher T.D., Ozono K., Michigami T., Tiosano D., Mughal M.Z., Mäkitie O. (2016). Global consensus recommendations on prevention and management of nutritional rickets. J. Clin. Endocrinol. Metab..

[84] Pludowski P., Marcinowska-Suchowierska E., Togizbayev G., Belaya Z., Grant W.B., Pilz S., Holick M.F. (2024). Daily and Weekly “High Doses” of Cholecalciferol for the Prevention and Treatment of Vitamin D Deficiency for Obese or Multi-Morbidity and Multi-Treatment Patients Requiring Multi-Drugs—A Narrative Review. Nutrients.

