# Supplementary material for: Expert Consensus on Optimizing the Strategy for the Prevention of Vitamin D Deficiency in Central Asia: From Scientific Evidence to Real-World Practice
Source: Nutrients. 2026 Jun 30;18(13):2122. doi: 10.3390/nu18132122 (PMC13363889; doi:10.3390/nu18132122)
Supplement: Supplementary file 1 [file nutrients-18-02122-s001.zip › nutrients-4361454-supplementary.pdf]

Supplementary Material File S1

Methodological Details of the Sun-Exposure Model for Cutaneous Vitamin D Synthesis in Central Asia

Purpose of the model

The sun-exposure model was developed as a practical, region-adapted tool to estimate the approximate duration of midday sun exposure required for cutaneous vitamin D synthesis in Central Asia. The model was intended to support public health communication and clinical counseling, rather than to replace individualized assessment of vitamin D status or laboratory measurement of serum 25(OH)D.

The model takes into account the geographical latitude of Central Asian cities, seasonal changes in solar elevation, the relative availability of UVB radiation, and the percentage of exposed body surface area. The model was designed to provide approximate estimates for preventive counseling in the general population.

General principle

Cutaneous vitamin D synthesis depends on the presence of UVB radiation reaching the skin. UVB availability is strongly influenced by latitude, season, time of day, atmospheric conditions, clothing, skin phototype, age, sunscreen use, and air pollution.

For the purpose of this model, effective vitamin D synthesis was assumed to occur primarily during the "solar window," when the sun elevation is sufficiently high above the horizon. A practical threshold of solar elevation >45° was used, corresponding approximately to the clinical "shadow rule," when a person's shadow is shorter than their height.

The model focused on midday exposure, as this is the period when UVB availability is highest.

Formula

The estimated monthly duration of sun exposure was calculated using the following formula:

$$T_{month} = T_{reference} / UVB_{coefficient}$$

where

Tmonth = estimated duration of sun exposure required in a given month;

Treference = reference exposure duration under optimal summer conditions;

UVBcoefficient = relative monthly UVB efficiency coefficient compared with the summer maximum.

The UVB coefficient was defined as a relative seasonal coefficient reflecting the expected reduction in effective UVB availability compared with peak summer conditions. A coefficient of 1.0 corresponds to the highest expected seasonal UVB efficiency, while lower values indicate progressively reduced UVB availability. A coefficient of 0 indicates that effective cutaneous vitamin D synthesis is unlikely or negligible under the defined assumptions.

Geographical zoning

Central Asia was divided into three practical latitude zones:

1. **Southern zone: 35–40° N latitude**

   Representative cities: Ashgabat, Dushanbe, southern areas of Uzbekistan and Tajikistan.

2. **Central zone: 41–45° N latitude**

Representative cities: Almaty, Bishkek, Tashkent.

3. **Northern zone: 46–55° N latitude**

Representative cities: Astana, Karaganda, Kostanay, Petropavlovsk.

This zoning was used because the required duration of sun exposure increases with latitude due to lower solar elevation and reduced seasonal UVB availability.

Reference exposure duration

Reference exposure duration was defined as the approximate duration of midday sun exposure required under favorable summer conditions, with 10–25% of body surface area exposed, in individuals without major risk factors impairing vitamin D synthesis.

The following reference values were used:

Southern zone: 20 minutes

Central zone: 25 minutes

Northern zone: 35 minutes

These values were selected based on published recommendations indicating that adults at mid-latitudes generally require approximately 15–30 minutes of midday sun exposure several times per week during summer when 10–25% of body surface area is exposed, with longer exposure required at higher latitudes, darker skin phototypes, older age, or lower exposed skin area.

Assumptions used in the model

The model was based on the following assumptions:

1. Sun exposure occurs near solar noon, within the period of highest UVB availability.
2. Approximately 10–25% of body surface area is exposed, corresponding to exposure of the face, arms, and lower legs.
3. Sunscreen is not applied during the short exposure period used for vitamin D synthesis.
4. The individual has a light-to-intermediate skin phototype. Individuals with darker skin phototypes may require longer exposure.
5. The individual is an adult without severe obesity, malabsorption, chronic kidney disease, chronic liver disease, granulomatous disease, or other disorders substantially affecting vitamin D metabolism.
6. The model does not account for individual variability in skin pigmentation, age, clothing habits, air pollution, cloud cover, altitude, or exact local UV index.
7. The model should not be used for infants and toddlers, for whom direct sun exposure is generally not recommended due to the risk of sunburn and long-term skin damage.
8. When the UVB coefficient is very low or zero, vitamin D synthesis is considered inefficient or negligible, and sun exposure should not be relied upon as a primary source of vitamin D.

Table S1. Relative monthly UVB efficiency coefficients used in the model

Month	Southern zone 35–40° N	Central zone 41–45° N	Northern zone 46–55° N
**January**	0	0	0
**February**	0.25	0	0
**March**	0.45	0.25	0
**April**	0.70	0.55	0.35
**May**	0.90	0.80	0.65
**June**	1.00	1.00	1.00
**July**	0.95	0.95	0.95
**August**	0.80	0.75	0.65
**September**	0.55	0.45	0.25
**October**	0.30	0.15	0
**November**	0	0	0
**December**	0	0	0

Interpretation of the UVB coefficient

The UVB coefficient is a simplified relative estimate of seasonal UVB efficiency. It was derived from the expected seasonal pattern of solar elevation and UVB radiation availability at different latitude bands. The coefficient is intended for practical approximation and visualization of seasonal differences, not for precise prediction of individual vitamin D synthesis.

Note: A coefficient below 0.25 indicates that cutaneous vitamin D synthesis is expected to be inefficient or unreliable. In such months, pharmacological supplementation and dietary sources of vitamin D should be prioritized.

Table S2. Estimated duration of midday sun exposure required for cutaneous vitamin D synthesis by latitude zone

Month	Southern zone 35–40° N	Central zone 41–45° N	Northern zone 46–55° N
**January**	Not effective	Not effective	Not effective
**February**	80 min	Not effective	Not effective
**March**	45 min	100 min	Not effective
**April**	30 min	45 min	100 min
**May**	25 min	30 min	55 min
**June**	20 min	25 min	35 min
**July**	20–25 min	25–30 min	35–40 min
**August**	25 min	35 min	55 min
**September**	35–40 min	55 min	140 min / not practical
**October**	65–70 min	165 min / not practical	Not effective
**November**	Not effective	Not effective	Not effective
**December**	Not effective	Not effective	Not effective

Table S3. Approximate sun-exposure estimates for selected Central Asian cities

City	Country	Latitude	Model zone	Favorable period	Summer exposure
**Ashgabat**	Turkmenistan	35–40° N	Southern zone	April–September	**20–25 min**
**Dushanbe**	Tajikistan	35–40° N	Southern zone	April–September	**20–25 min**
**Tashkent**	Uzbekistan	41–45° N	Central zone	April–September	**25–30 min**
**Bishkek**	Kyrgyz Republic	41–45° N	Central zone	April–September	**25–30 min**
**Almaty**	Kazakhstan	41–45° N	Central zone	April–September	**25–30 min**
**Astana**	Kazakhstan	46–55° N	Northern zone	May–August	**35–40 min**
**Karaganda**	Kazakhstan	46–55° N	Northern zone	May–August	**35–40 min**
**Kostanay**	Kazakhstan	46–55° N	Northern zone	May–August	**35–40 min**
**Petropavlovsk**	Kazakhstan	46–55° N	Northern zone	May–August	**35–40 min**

Practical interpretation

The model suggests that in the southern and central parts of Central Asia, cutaneous vitamin D synthesis is most feasible from spring to early autumn, particularly during midday hours. In northern Kazakhstan, the effective period is shorter and is mostly limited to late spring and summer.

Important: During late autumn, winter, and early spring, particularly in cities located at or above 41–45° N latitude, cutaneous vitamin D synthesis becomes inefficient or negligible. During these months, vitamin D status should be maintained primarily through prophylactic supplementation and dietary sources rather than sun exposure.

For individuals with darker skin phototypes, older age, obesity, limited mobility, closed clothing, sunscreen use, or reduced exposed body surface area, the required exposure duration may be substantially longer than the estimates provided in this model.

Model limitations and implementation considerations

This model is intended as a simplified public health and clinical counseling tool. It does not provide individualized estimates of vitamin D synthesis and should not be used as a substitute for serum 25(OH)D testing in patients with confirmed deficiency, high-risk conditions, or disorders affecting vitamin D metabolism.

The estimates may be influenced by local weather, cloud cover, altitude, air pollution, clothing habits, exact UV index, and skin phototype. Therefore, the model should be interpreted as approximate and should be updated when more detailed regional UVB and population data become available.

Future work should include validation of the model using measured UV index data, city-specific solar radiation datasets, regional clothing and lifestyle patterns, and serum 25(OH)D measurements across different seasons in Central Asian populations.
